# Improved sugar yields from biomass sorghum feedstocks: comparing low-lignin mutants and pretreatment chemistries

**DOI:** 10.1186/s13068-016-0667-y

**Published:** 2016-11-21

**Authors:** Bruno Godin, Nick Nagle, Scott Sattler, Richard Agneessens, Jérôme Delcarte, Edward Wolfrum

**Affiliations:** 1 National Bioenergy Center, National Renewable Energy Laboratory (NREL), 15013 Denver West Parkway, Golden, CO 80401 USA; 2 Valorization of Agricultural Products Department-Biomass, Bioproducts and Energy Unit, Walloon Agricultural Research Center (CRA-W), Chaussée de Namur, 146, 5030 Gembloux, Belgium; 3 Agricultural Research Service (ARS)-Grain, Forage, and Bioenergy Research Unit, United States Department of Agriculture (USDA), University of Nebraska, 251 Filley Hall/Food Industries Complex, East Campus, Lincoln, NE 68583-0937 USA

**Keywords:** *Sorghum bicolor*, *Brown midrib*, Pretreatment, Total sugar yield, Biofuels

## Abstract

**Background:**

For biofuel production processes to be economically efficient, it is essential to maximize the production of monomeric carbohydrates from the structural carbohydrates of feedstocks. One strategy for maximizing carbohydrate production is to identify less recalcitrant feedstock cultivars by performing some type of experimental screening on a large and diverse set of candidate materials, or by identifying genetic modifications (random or directed mutations or transgenic plants) that provide decreased recalcitrance. Economic efficiency can also be increased using additional pretreatment processes such as deacetylation, which uses dilute NaOH to remove the acetyl groups of hemicellulose prior to dilute acid pretreatment. In this work, we used a laboratory-scale screening tool that mimics relevant thermochemical pretreatment conditions to compare the total sugar yield of three near-isogenic *brown midrib* (*bmr*) mutant lines and the wild-type (WT) sorghum cultivar. We then compared results obtained from the laboratory-scale screening pretreatment assay to a large-scale pretreatment system.

**Results:**

After pretreatment and enzymatic hydrolysis, the *bmr* mutants had higher total sugar yields than the WT sorghum cultivar. Increased pretreatment temperatures increased reactivity for all sorghum samples reducing the differences observed at lower reaction temperatures. Deacetylation prior to dilute acid pretreatment increased the total sugar yield for all four sorghum samples, and reduced the differences in total sugar yields among them, but solubilized a sizable fraction of the non-structural carbohydrates. The general trends of increased total sugar yield in the *bmr* mutant compared to the WT seen at the laboratory scale were observed at the large-scale system. However, in the larger reactor system, the measured total sugar yields were lower and the difference in total sugar yield between the WT and *bmr* sorghum was larger.

**Conclusions:**

Sorghum *bmr* mutants, which have a reduced lignin content showed higher total sugar yields than the WT cultivar after dilute acid pretreatment and enzymatic hydrolysis. Deacetylation prior to dilute acid pretreatment increased the total sugar yield for all four sorghum samples. However, since deacetylation also solubilizes a large fraction of the non-structural carbohydrates, the ability to derive value from these solubilized sugars will depend greatly on the proposed conversion process.

**Electronic supplementary material:**

The online version of this article (doi:10.1186/s13068-016-0667-y) contains supplementary material, which is available to authorized users.

## Background

Sorghum (*Sorghum bicolor* L. Moench) is an important grain and forage crop around the world. The interest in sorghum as an important potential source of biomass for biofuel and biochemical productions has also been growing because of its availability and sustainability [1, 2]. Sorghum is an annual tropical grass that fixes carbon through C4 photosynthesis. This crop is easily established, tolerant to drought, has low water needs, efficiently uses nutrients, has high dry matter harvest yields, is digestible for livestock, and is economical to produce. Sorghum is often grown in areas that are too hot and dry for corn [1, 2].

To improve its digestibility for cattle while maintaining similar dry matter harvest yields, *brown midrib* (*bmr*) sorghum mutants were isolated from chemically mutagenized populations. Both *bmr6* and *bmr12* mutants are available in commercial sorghum lines [2]. The *bmr6* gene encodes a cinnamyl alcohol dehydrogenase (CAD2) enzyme while the *bmr12* gene encodes for a caffeic *O*-methyltransferase (COMT) enzyme. The alleles of *bmr6* and *bmr12*, respectively, are nonsense mutations, and are likely “null alleles,” because stop codons are predicted to prematurely truncate the respective peptide prior to conserved catalytic domain of the proteins. Their respective transcripts and protein are nearly undetectable [3, 4]. These *bmr* mutants have an improved digestibility for ruminants because of their reduced lignin content [2] although some work has shown slightly lower harvest yields [5]. Lignin is a phenylpropane macromolecule found in the cell walls of all vascular plants. It is essential to those plants because it provides them mechanical and structural rigidity and protects them from abiotic and biotic stresses. Lignin also forms a barrier surrounding the plants’ polysaccharides: cellulose, hemicelluloses, and pectins and thus it inhibits the enzymatic hydrolysis of plant cell wall polysaccharides in the rumen as well as in bioconversion processes for biofuel and biochemical production [5, 6]. Indeed, we have shown a strong negative correlation between the lignin content and the total sugar yields from structural of glucan and xylan in a laboratory-scale dilute acid pretreatment and enzymatic hydrolysis assay for a wide variety of herbaceous biomass feedstocks including sorghum [7].

The bioconversion of cellulosic biomass to biofuels typically consists of three distinct steps: pretreatment, enzymatic hydrolysis, and fermentation. The first two steps enable the release of monomeric sugars from the structural carbohydrates in the biomass, while the third step is the microbial conversion of the released sugars (mainly glucose and xylose) to the desired biofuel (e.g., ethanol and butanol). For a biofuel production process to be economically efficient, it must be able to produce and then convert soluble monomeric sugars from the structural glucan and xylan of the biomass [8–10]. Therefore, it is essential to identify less recalcitrant biomass feedstocks and develop a process to enhance the reactivity of these feedstocks while reducing inputs (e.g., energy and chemicals). Less recalcitrant feedstocks can be identified by screening diverse panel plant cultivars [7] or through genetic modifications that affect lignin synthesis, such as the *bmr* mutants described above. The thermal, chemical, and physical conditions of the pretreatment step enable us to open up the cell wall structure to expose the glucan and xylan to the enzymes of the enzymatic hydrolysis step [11]. Preprocessing biomass can improve the reactivity of the biomass prior to the thermochemical pretreatment [12]. For example, deacetylation with dilute sodium hydroxide (NaOH) removes the acetyl groups from the xylan backbone of the hemicelluloses. Soluble components such as ash, protein, and non-structural carbohydrates are also removed, enriching the glucan and xylan fractions. The deacetylation is typically performed at NaOH concentrations of 0.2–0.4% (w/w) and temperatures of 60–80 °C for 30–180 min [13, 14]. Removing the acetyl groups improves the total sugar yield of the biomass during the enzymatic hydrolysis step and also reduces the concentration of fermentation inhibitors such as acetic acid in the enzymatic hydrolyzate [13]. Studies are available on the deacetylation of corn, but not yet on sorghum. One of those studies shows a strong negative correlation between the degree of acetylation of the corn stover biomass and the enzymatic hydrolysis yield [15].

The purpose of this work is to compare the sugar yields of wild-type (WT) sorghum to its near-isogenic *bmr* lines, which have reduced lignin content, using a laboratory-scale deacetylation and dilute acid pretreatment and enzymatic hydrolysis assay. This work builds on the work of [6], which examined the dilute acid pretreatment of sorghum *bmr* mutants and WT cultivars. In this work, we use a similar sorghum variety (AWheatland) for which the *bmr* sorghum mutants are known to have similar biomass yields compared to WT [2, 3, 16]. We extend this work by examining a wider variety of dilute acid pretreatment conditions, by evaluating the effect of deacetylation prior to dilute acid pretreatment on the total sugar yield from these sorghum samples, and by comparing the relative performance of the sorghum mutants from this laboratory-scale assay with the relative performance in a larger and more process-relevant pretreatment reactor.

## Results and discussion

### A note on total sugar yield calculations

For this study, the total sugar yield of the feedstocks is expressed as the combined yield of glucose and xylose from the corresponding structural carbohydrates glucan and xylan. A yield calculation is typically a ratio, with the numerator containing the mass of product released, and the denominator containing the mass of reactant originally present. For this work, the yield numerator is the sum of glucose and xylose released in their monomeric or oligomeric forms by all pretreatments (deacetylation and/or dilute acid pretreatment) and the monomeric glucose and xylose released by enzymatic hydrolysis. The denominator is the glucan and xylan content contained in the feedstock (including the anhydro correction factor) prior to any pretreatment or enzymatic hydrolysis. However, the presence of non-structural carbohydrates (free glucose, sucrose, and starch) in the feedstock elevates the apparent glucose yield. There are two ways to account for the presence of the non-structural derived glucose in the calculated yield:

1. Adding the non-structural glucose (expressed in its monomeric form) to the denominator (DE) of the yield. This will correspond to the total glucose and xylose yield from both structural and non-structural carbohydrates. This yield expression focuses on the availability of all potentially fermentable carbohydrates.
2. Subtracting the non-structural glucose content (expressed in its monomeric form) from the numerator (NU) of the yield. This assumes all non-structural carbohydrates are converted to glucose, and makes the resulting yield calculation correspond to the glucose and xylose yield from only structural carbohydrates. This yield expression focuses on the sugar yield from the structural carbohydrates of the feedstock.

Either method is valid, although there might be compelling reasons to use one calculation over the other, depending on the specific research question to be answered. Regardless, it is important to keep in mind how the total sugar yield is calculated. In this work, we use the DE method, and focus on the availability of all potentially fermentable carbohydrates. We present yields calculated with both the DE and NU method in Additional file 1.

The fate of the non-structural carbohydrates (principally glucose) released during deacetylation should also be considered with caution. In the current state of cellulosic conversion technology, the deacetylation stream is burned for energy, and any solubilized carbohydrates in this stream cannot be recovered [14]. For the glucose and xylose yields in this work, we define the “no-loss recovery” (NL) to include all sugars solubilized during deacetylation and the “loss recovery” (LO) to exclude all sugars solubilized during deacetylation. We compare the two calculations when we assess the impact of deacetylation on the total sugar yield.

### Feedstock compositional analysis

The chemical composition [glucan, xylan, galactan, arabinan, acetyl, acid-soluble lignin, insoluble (Klason) lignin, total lignin, starch, total soluble sugars, water extractives, ethanol extractives, proteins, and mineral compounds] of the near-isogenic sorghum lines [wild type (WT), *bmr6* mutant, *bmr12* mutant, and *bmr6 bmr12* stacked mutant (SM)] are shown in Table 1. The non-structural carbohydrate content (total soluble sugars and starch) of the analyzed sorghum feedstocks represented nearly 20% of their mass. Thus, the glucose released from those non-structural carbohydrates can be expected to have a significant effect on the total glucose yield, as we will show later in this report.

**Table 1:** Chemical composition of the sorghum feedstocks (g/g TS)

	Wild type	Stacked mutant	*bmr6* mutant	*bmr12* mutant
Glucan	0.243 ± 0.007a	0.252 ± 0.010a	0.226 ± 0.002b	0.253 ± 0.001a
Xylan	0.144 ± 0.004b	0.150 ± 0.005b	0.135 ± 0.001c	0.163 ± 0.001a
Galactan	0.0110 ± 0.0003b	0.0112 ± 0.0005ab	0.0114 ± 0.0004ab	0.0119 ± 0.0005a
Arabinan	0.0255 ± 0.0009bc	0.0266 ± 0.0012ab	0.0246 ± 0.0003c	0.0277 ± 0.0003a
Acetyl	0.0312 ± 0.0007a	0.0227 ± 0.0003b	0.0182 ± 0.0002d	0.0200 ± 0.0002c
Soluble Klason lignin	0.0095 ± 0.0007a	0.0105 ± 0.0025a	0.0106 ± 0.0002a	0.0097 ± 0.0009a
Insoluble Klason lignin	0.103 ± 0.002a	0.0833 ± 0.0010d	0.0880 ± 0.0009c	0.0967 ± 0.0016b
Total Klason lignin	0.113 ± 0.002a	0.0939 ± 0.0030d	0.0985 ± 0.0009c	0.106 ± 0.001b
Starch	0.0313 ± 0.0015a	0.0286 ± 0.0002a	0.0229 ± 0.0013b	0.0250 ± 0.0007b
Total soluble sugars	0.153 ± 0.001c	0.158 ± 0.001b	0.169 ± 0.002a	0.124 ± 0.001d
Water extractives	0.285 ± 0.020b	0.290 ± 0.027b	0.338 ± 0.005a	0.260 ± 0.006b
Ethanol extractives	0.0327 ± 0.0008ab	0.0321 ± 0.0007b	0.0329 ± 0.0013ab	0.0342 ± 0.0002a
Protein	0.0506	0.0547	0.0543	0.0534
Mineral compounds	0.0929 ± 0.0007b	0.0881 ± 0.0022c	0.0989 ± 0.0012a	0.0931 ± 0.0028b

All samples analyzed in triplicate except for starch (duplicate) and protein (singlet). Total soluble sugars = sucrose + free glucose + free fructose. The uncertainty corresponds to 95% confidence interval of the mean. For each chemical compound, sorghum feedstocks with the same letter are not significantly different with the Tukey–Kramer multiple comparison test

The detergent fiber composition (neutral detergent fiber (NDF), acid detergent fiber (ADF), acid detergent lignin (ADL), calculated cellulose (ADF-ADL), hemicellulose (NDF-ADF), and enzymatically digestible organic matter (eDOM) of the analyzed sorghum feedstocks are shown in Table 2. There were significant differences in total lignin, ADL, and eDOM among these four sorghums. The *bmr* sorghum lines compared to the WT had a reduced total lignin and ADL and an increased eDOM for all three lines. For total lignin, the reduction varies from 6 to 17%. For ADL, the reduction varies from 21 to 50%. For eDOM, the increase varies from 6 to 14%. Interestingly, the total lignin, ADL, and eDOM changes seem to be additive in the mutants when combined into the SM, which was also observed for total lignin and ADL in similar *bmr* materials [6]. The increase of the eDOM for *bmr* sorghums confirms that they are feedstocks with an improved digestibility for ruminants, likely because eDOM is highly correlated to the lignin content [17–19]. The *bmr* sorghum lines also had a significantly reduced acetyl content of at least 27% compared to the WT. A reduced lignin and acetyl content should increase the total sugar yield of the biomass from the *bmr* lines. The other structural components determined (glucan, xylan, NDF, ADF, cellulose Van Soest, and hemicelluloses Van Soest) were also affected by the *bmr* mutations.

**Table 2:** Detergent fibers composition (g/g TS) and enzymatically digestible organic matter (g/g VS) of the sorghum feedstocks

	Wild type	Stacked mutant	*bmr6* mutant	*bmr12* mutant
Neutral detergent fiber (NDF)	0.526 ± 0.002b	0.504 ± 0.007c	0.487 ± 0.005d	0.541 ± 0.007a
Acid detergent fiber (ADF)	0.277 ± 0.002b	0.267 ± 0.003bc	0.264 ± 0.001c	0.299 ± 0.010a
Acid detergent lignin (ADL)	0.0186 ± 0.0016a	0.0092 ± 0.0017c	0.0119 ± 0.0007bc	0.0147 ± 0.0010b
Cellulose Van Soest (ADF-ADL)	0.259 ± 0.001b	0.258 ± 0.002b	0.252 ± 0.001c	0.280 ± 0.001a
Hemicellulose Van Soest (NDF-ADF)	0.249 ± 0.004a	0.237 ± 0.008b	0.223 ± 0.005c	0.242 ± 0.004ab
Enzymatically digestible organic matter (eDOM)	0.590 ± 0.006d	0.675 ± 0.003a	0.643 ± 0.006b	0.624 ± 0.003c

All analyses performed in triplicate. The uncertainty corresponds to 95% confidence interval of the mean. For each chemical compound, sorghum feedstocks with the same letter are not significantly different with the Tukey–Kramer multiple comparison test

The detergent fiber method is less reliable for estimating structural components (cellulose, hemicelluloses, and lignin) compared to the dietary fiber method [20]. For a feedstock such as sorghum, the detergent fiber method compared to the dietary fiber method overestimates cellulose and hemicelluloses, and underestimates lignin, as observed in the present study (Tables 1, 2) [20–23]. The ADL values were especially low, but they were consistent with those values observed by [6], which used the same *bmr* mutants in a different variety of sorghum. The chemical composition and detergent fiber composition analyses of the present study are consistent with that study; the main differences were that the sorghum samples of [6] contained much less mineral content and, therefore, had slightly higher concentrations of the other compounds compared to the results of Tables 1 and 2. In addition, the glucan, xylan, NDF, and ADF contents of the *bmr6* mutant of the present study are lower compared to those of [6]. While these lines contain the same two *bmr* mutations, they are in a different genetic background. We used the “A Wheatland” variety, whereas the earlier work used the “Atlas” sorghum variety.

### Total sugar yield: optimizing dilute acid pretreatment

**Fig. 1:**
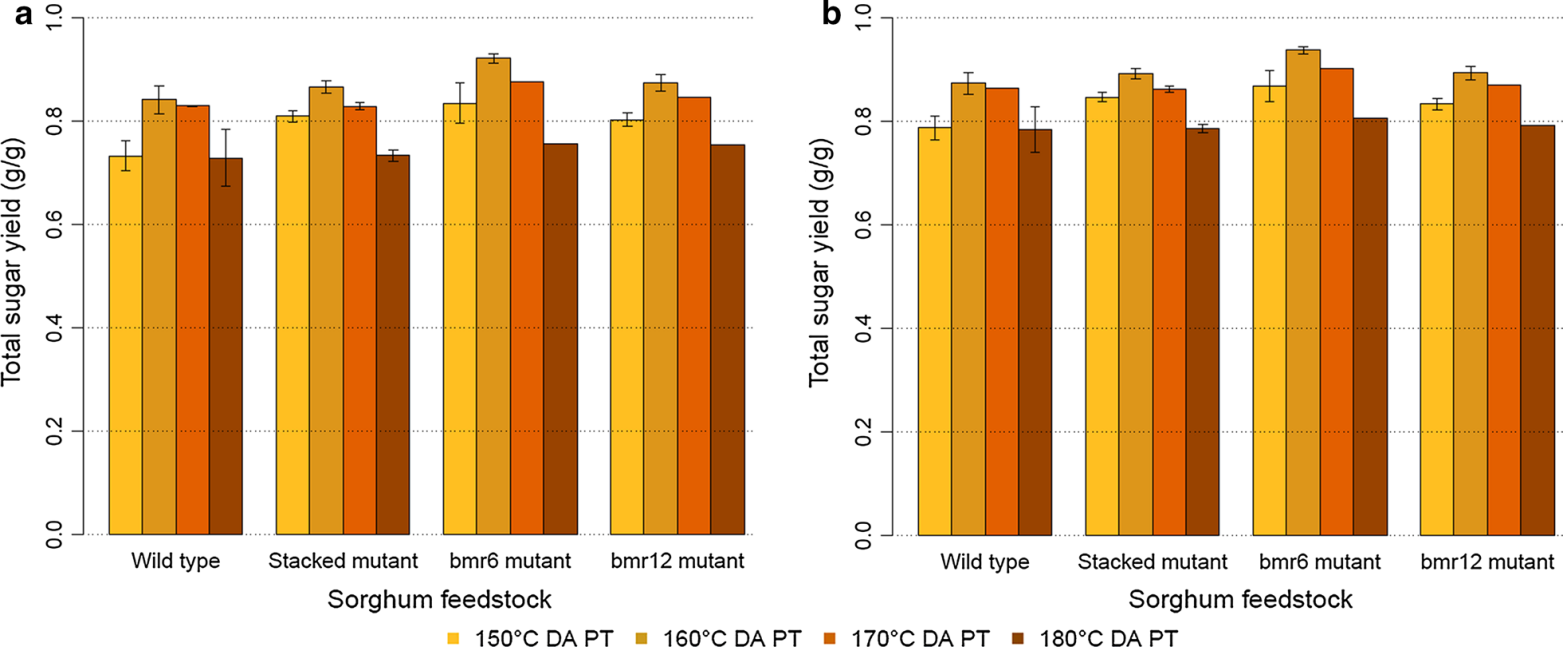
Total sugar yield (g/g) of sorghums with dilute acid (DA) pretreatment (PT) on the ASE350 reactor and enzymatic hydrolysis. **a** Glucose and xylose yield from structural carbohydrates (NU). **b** Glucose and xylose yield from structural and non-structural carbohydrates (DE). *Error bars* correspond to 95% confidence interval of the mean

The total sugar yields (combined glucose and xylose yield) of the near-isogenic sorghum lines with dilute acid pretreatment at different temperatures (150, 160, 170, and 180 °C), followed by enzymatic hydrolysis are illustrated in Fig. 1a, b. How the non-structural carbohydrates were accounted for in the calculation of total sugar yield (either subtracting the non-structural carbohydrates from the numerator, NU or adding them to the denominator, DE) has a modest impact on the calculated total sugar yield, with the DE calculation having calculated total sugar yields 2–8% higher (ANOVA *p* value <0.001) compared to total sugar yields from the NU calculation (data not shown). The difference is minor because the sugars in the dilute acid pretreatment liquor, regardless of their origin, are included in the total sugar yield. Note that the primary analytical data used for both of these calculations are identical; the differences in yields are due simply to how the total sugar yield calculation is performed. As mentioned above, we present all total sugar yields in this work using the DE calculation, focusing on the total soluble carbohydrate yield from both structural and non-structural carbohydrates.

The total sugar yields of these sorghum feedstocks show the same trend with dilute acid pretreatment temperature as the feedstocks analyzed by [7]. Both the pretreatment temperature and the sorghum mutant have significant impacts (ANOVA *p* value <0.001) on the total sugar yield (Table 3). Figure 1a, b show that the optimal pretreatment temperature for all four sorghum feedstocks is approximately 160 °C. Above 160 °C, these feedstocks are “overcooked” and the total sugar yield drops (data included in Additional file 1). The total sugar yield of the SM is significantly higher than the WT (ANOVA *p* value <0.001, Table 3) at 150 °C, but not at the higher temperatures. Increasing the dilute acid pretreatment severity decreases the difference in recalcitrance between the feedstocks. This is supported by the ANOVA results in Table 3 as well, which shows a significant second-order interaction between feedstock type and temperature.

**Table 3:** ANOVA of the total sugar yield (g/g) of the sorghum feedstocks from ASE350 experiments

Effect	Glucose and xylose yield from structural and non-structural carbohydrates, and recovery of carbohydrates solubilized during deacetylation (DE-NL)		Glucose and xylose yield from structural and non-structural carbohydrates, and loss recovery of carbohydrates solubilized during deacetylation (DE-LO)	
	*F* value	*p* value	*F* value	*p* value
Feedstock	60.98	<10^−5^	56.44	<10^−5^
Temperature	79.91	<10^−5^	81.07	<10^−5^
Deacetylation	71.96	<10^−5^	758.1	<10^−15^
Batch	0.566	0.46	1.183	0.28
Temperature * feedstock	7.368	0.0096	8.170	0.0067
Feedstock * deacetylation	0.003	0.95	0.198	0.66
Temperature * deacetylation	17.16	0.0002	17.89	0.0001
Dilute acid pretreatment temperature at 150 °C				
Feedstock	46.54	<10^−5^	44.87	<10^−5^
Deacetylation	66.97	<10^−5^	226.6	<10^−5^
Batch	0.207	0.65	0.479	0.50
Feedstock * deacetylation	0.790	0.39	1.154	0.30
Dilute acid pretreatment temperature at 160 °C				
Feedstock	15.74	0.0008	13.07	0.0018
Deacetylation	11.43	0.0031	608.7	<10^−15^
Batch	0.391	0.54	0.734	0.40
Feedstock * deacetylation	0.958	0.34	0.358	0.55

Only experimental data from pretreatment reactor temperatures of 150 and 160 °C are included in this analysis, since higher pretreatment temperatures caused excessive degradation of solubilized xylose (see text)

The total sugar yields calculated in this work (again, using the DE method), are higher than the results of [6], which likely used yields calculated in the same manner. These differences are expected since [6] used a lower dilute acid pretreatment temperature (121 °C) leading to lower conversions. We note that [6] observed a slightly larger improvement of the total sugar yield for the stacked mutant (SM) material compared to the WT cultivar. This may be an artifact of the differences in experimental systems, or a fundamental difference in the two sorghum cultivars used in these two works.

As noted in the “Methods” section, both monomeric and oligomeric sugars are released during the dilute acid pretreatment. Sugars in either form contribute to the total sugar yield. While more severe pretreatment conditions (e.g., longer times, higher temperatures, or higher acid concentrations) result in increased monomeric sugar yield and reduced oligomeric sugar yield, we did not observe any systematic difference in monomeric versus oligomeric sugar yield from the sorghum mutants studied in this work.

### Total sugar yield: effect of deacetylation

**Fig. 2:**
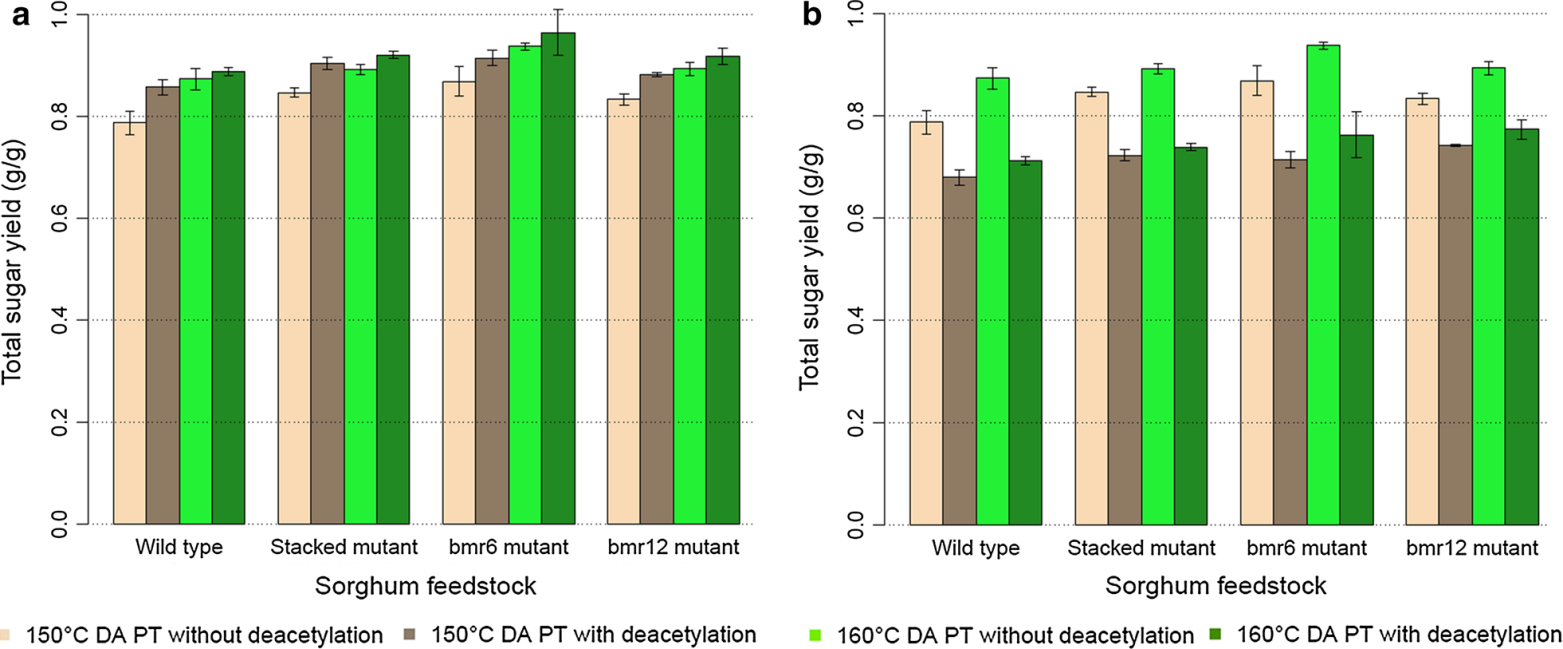
Total sugar yield (g/g) of the sorghums with or without deacetylation on the ASE350 reactor and enzymatic hydrolysis. **a** Glucose and xylose yield from structural and non-structural carbohydrates, and no-loss recovery of carbohydrates solubilized during deacetylation (DE-NL). **b** Glucose and xylose yield from structural and non-structural carbohydrates, and loss recovery of carbohydrates solubilized during deacetylation (DE-LO). *Error bars* correspond to 95% confidence interval of the mean. *DA* dilute acid, *PT* pretreatment

We examined the effect of deacetylation prior to dilute acid pretreatment for dilute acid pretreatment temperatures of 150 and 160 °C only, since higher dilute acid pretreatment temperatures were clearly non-optimal. The results of these experiments are shown in Fig. 2 and Table 2. The total sugar yields presented in Fig. 2a assume all sugars solubilized during deacetylation are not lost during the deacetylation step while the yields presented in Fig. 2b assume that any solubilized sugars are lost during deacetylation. Deacetylation and increased dilute acid pretreatment temperature significantly increase the total sugar yield for all feedstocks, and the SM is more reactive than the WT (ANOVA *p* value <0.001, Table 3). For the DE-LO calculation, there is a significant (ANOVA *p* value <0.001) decrease (between 14 and 19%) of the total sugar yield due to deacetylation. This can be explained by the high content of non-structural carbohydrates in the analyzed sorghums (Table 1), since the DE-LO calculation assumes that non-structural carbohydrates (total soluble sugars and a fraction of starch) solubilized during deacetylation are lost prior to enzymatic hydrolysis.

A major aim of the deacetylation process is to solubilize the acetyl groups from the xylan chains. This solubilization was significantly (ANOVA *p* value <0.001) higher in *bmr* mutants (between 0.33 and 0.41 g/g) compared to the WT (0.23 g/g) (Table 4). The increased acetyl removal likely contributed to the higher total sugar yield of the deacetylated sorghums.

**Table 4:** Glucose, xylose, and acetyl yield (g/g) of the sorghum feedstocks by deacetylation

	Wild type	Stacked mutant	*bmr6* mutant	*bmr12* mutant
Glucose^a^	0.245 ± 0.002	0.250 ± 0.001	0.279 ± 0.005	0.199 ± 0.002
Xylose	0.0222 ± 0.0011	0.0240 ± 0.0010	0.0261 ± 0.0012	0.0249 ± 0.0010
Acetyl	0.230 ± 0.008	0.331 ± 0.014	0.412 ± 0.014	0.412 ± 0.005

The uncertainty corresponds to 95% confidence interval of the mean

^a^Glucose refers to total glucose (non-structural and structural)

If we assume recovery of soluble sugars, deacetylation improves the total sugar yield for all feedstocks and both dilute acid pretreatment temperatures. However, the positive effect of deacetylation is less pronounced at the higher dilute acid pretreatment temperature, and the difference in total sugar yields between the mutants and the WT cultivar also diminish at the higher dilute acid pretreatment temperature. That is, the higher dilute pretreatment temperature increases the total sugar yield, but reduces the differences in recalcitrance among feedstocks and the effect of additional preprocessing using deacetylation. Again, these observations are supported by the statistically significant second-order effects in the ANOVA (Table 3).

### Total sugar yield: ZipperClave reactor

**Fig. 3:**
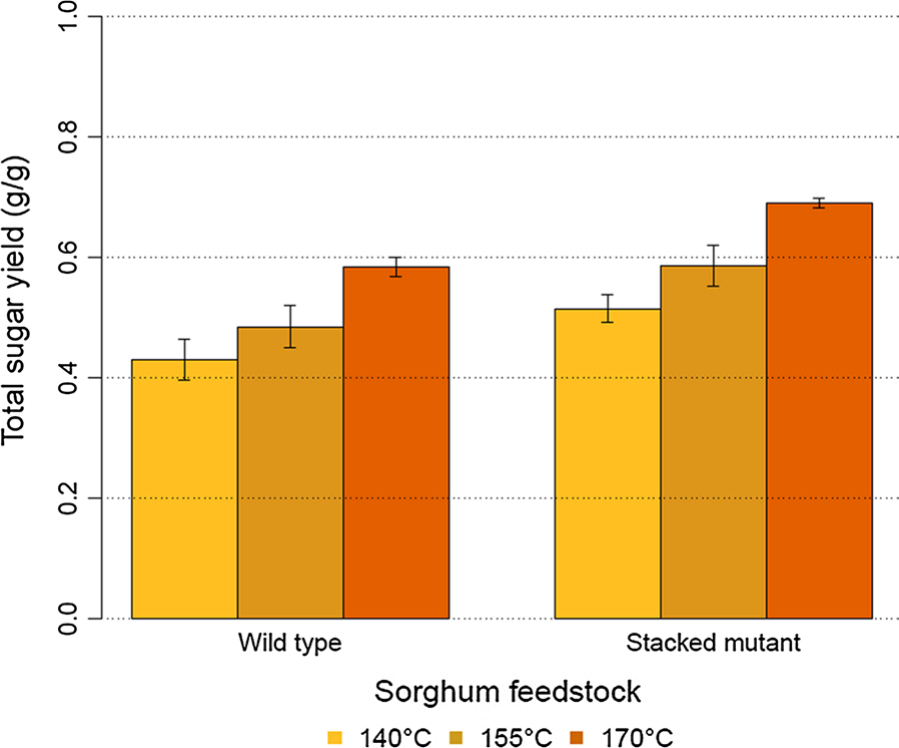
Total sugar yield (g/g) of sorghums with dilute acid (DA) pretreatment (PT) on the ZipperClave reactor and enzymatic hydrolysis. Glucose and xylose yield from structural and non-structural carbohydrates (DE). *Error bars* correspond to 95% confidence interval of the mean

We examined the behavior of the WT sorghum and the SM *bmr* mutant in the larger ZipperClave pretreatment reactor. The total sugar yield for the SM was higher than the WT for all three pretreatment temperatures investigated (Fig. 3). However, the measured total sugar yields from the ZipperClave experiments were lower than the corresponding total sugar yields determined using the ASE350 reactor system. This suggests that other factors, such as the method of heating (steam vs. electrical), mixing, heat transfer, and reactor configuration may have affected the pretreatment conversion in the ZipperClave reactor. Nonetheless, we saw consistent experimental results with both systems; higher dilute acid pretreatment temperatures increase total sugar yield, and a large improvement in total sugar yield with the SM mutant.

## Conclusions

Using a laboratory-scale screening tool working at relevant biofuel process conditions, we assessed the total sugar yields of near-isogenic *bmr* sorghum mutants with reduced lignin content and their WT under different pretreatment conditions. Deacetylation before dilute acid pretreatment, *bmr* mutants compared to WT, and the higher temperature of dilute acid pretreatment significantly increased the total sugar yield. These differences decreased as the dilute acid pretreatment temperature increased.

We do not yet understand the ultimate origin of the differences in recalcitrance among the sorghum cultivars tested in this work. There are known differences in the lignin structures of the mutants. The lignin S/G ratio for the *bmr12* mutant is ~0.03, for the *bmr6* it is ~0.3 and for the wild type it is ~0.6 [24, 25]. However, since we found that the *bmr6* mutant rather than the bmr12 mutant was the least recalcitrant of all four cultivars, the S/G ratio is clearly not the controlling factor. More work is needed to understand the structural differences among these mutants causing the differences in measured recalcitrance.

How non-structural carbohydrates are accounted for in the total sugar yield calculation in feedstocks with high non-structural carbohydrates content (e.g., the DE vs. NU calculation) has a modest impact on the calculated yields, with higher calculated yields if they are added to the denominator (DE) of the yield calculation compared to when they are subtracted from the numerator of the yield (NU). However, how non-structural sugars that are solubilized during deacetylation are accounted for in feedstocks with high non-structural carbohydrate content greatly affects the calculated total sugar yield, with the “no-loss” yield calculation providing much higher yields than the “loss” calculation. The choice of calculation should be based on the intended process concept. If the carbohydrates solubilized during deacetylation are not recovered then feedstocks with high non-structural carbohydrates should not undergo deacetylation prior to dilute acid hydrolysis.

The results of the ASE350 small-scale laboratory pretreatment and the larger ZipperClave system showed similar trends for increased total sugar yield with temperature and for the SM *bmr* mutant compared to the WT. The differences in reactor profile, heating, and heat transfer between the two systems present challenges in accurately mapping smaller systems to larger pretreatment systems.

## Methods

### Sorghum feedstock samples

The stover was collected from four near-isogenic sorghum lines (*S. bicolor* L. Moench) (grain heads removed): WT, *bmr6* mutant, *bmr12* mutant, and stacked (both *bmr6 bmr12 mutations)* mutant (SM) A Wheatland [26, 27]. The plants were grown in 2012 at the University of Nebraska Field Laboratory, Ithaca, NE (Sharpsburg silty clay loam, fine, smectitic, mesic Typic Argiudoll). Nitrogen fertilizer was applied prior to planting at 112 kg/ha. Individual plots consisted of two 7.6-m rows spaced 76 cm apart. Materials were planted on 17 May, 2012, and a pre-emergent application of atrazine (1.1 kg/ha) was applied on 18 May, 2012. There were five irrigation applications: 9 (3.8 cm), 17 (3.2 cm), 25 (3.2 cm), 30 July (3.2 cm), and 29 August (3.2 cm), 2016. The plots (row) were harvested with a commercial forage harvester (New Holland Model 718) on 10 September, 2012. The samples were oven-dried at 60 °C to less than 10% moisture and milled with a 2 mm screen knife mill (Thomas Scientific, Swedesboro, NJ, USA).

### Compositional analysis

The appropriate National Renewable Energy Laboratory (NREL) laboratory analytical procedures (LAP) were used to determine the sugar and organic acid concentrations of the compositional analysis, pretreatment, and enzymatic hydrolysis experiments [28–30]. One exception to these procedures was that (as mentioned above) the samples were oven-dried at 60 °C rather than at or below 45 °C. All samples were dried in the same manner.

The neutral detergent fibers (NDF: weight of the neutral detergent fiber residue corrected for mineral compounds), acid detergent fibers (ADF: weight of the acid detergent fiber residue corrected for mineral compounds), and acid detergent lignin (ADL: weight of the acid detergent lignin residue corrected for mineral compounds) were determined by the Van Soest gravimetric method [31, 32].

The enzymatically digestible organic matter (eDOM) was determined by the De Boever method [33]. The eDOM allows us to assess the digestibility of feedstocks for ruminants. Briefly, samples were incubated, in chronological order, with pepsin in 0.1 M HCl for 24 h at 40 °C, with 0.1 M HCl for 45 min at 80 °C, and with cellulase in an acetate buffer at pH 4.8 for 24 h at 40 °C.

### Pretreatment assay

For the smaller bench-scale assay (ASE350 accelerated solvent extractor; Dionex, Sunnyvale, CA, USA), each pretreatment experiment condition was performed in two batches of triplicates for WT and SM, and in two batches of one replicate for *bmr6* and *bmr12* mutants, except for the experimental conditions of dilute acid pretreatment at 170 and 180 °C, where only one batch of triplicates and one batch of one replicate was done for WT and SM, and for *bmr6* and *bmr12* mutants, respectively. More replicates have been performed on the WT and SM to be able to assess with more statistical power the difference between the most distinctive analyzed feedstocks.

For the large-scale assay (ZipperClave^®^ reactor), the experiments were performed only for WT and SM. They were performed in three batches of one replicate.

### ASE350 acid pretreatment

The smaller bench-scale assay was performed using an ASE350 accelerated solvent extractor (Dionex, Sunnyvale, CA, USA) following the procedure of [7], except that the experiments used 4.0 g of dry biomass and 40 mL of 1% (w/w) sulfuric acid for the dilute acid pretreatment step, and 133 mL of deionized (DI) water for the rinse step. The dry solids loading for the dilute acid pretreatment step remained at approximately 10% (w/w). Briefly, a 66 mL zirconium cell was used as a reaction vessel and the liquor was collected in a 250 mL glass bottle. The pretreatment step consisted of a 7 min heating period followed by 6 min static time at a temperature between 150 and 180 °C, which was fixed depending on the experiment. The cell temperature was then reduced to 100 °C before proceeding to the water rinse step. At the end of the assay, the liquor in the glass bottle was transferred quantitatively to a 200 mL volumetric flask, which was brought to volume with DI water.

The washed solid and an aliquot of the liquor were both transferred in separate 50 mL plastic tubes. They were stored in a refrigerator until further analysis. An aliquot of the liquor was hydrolyzed at 4% (w/w) sulfuric acid for 1 h at 121 °C in an autoclave, neutralized with calcium carbonate, then filtered, and analyzed for total monomeric sugars and organic acids. An aliquot of the washed solid was used to determine its solid content after one night of drying in an oven at 103 °C. This last analysis was started 1 day before the washed solid underwent enzymatic hydrolysis.

This assay uses the total amount of sugars (both in monomeric and oligomeric form) released during pretreatment as part of the total sugar yield. More severe pretreatments typically result in higher monomeric sugar release and lower oligomeric sugar release. Only monomeric sugars are measured after enzymatic hydrolysis; our experience has shown no oligomeric sugars present after the EH assay.

### ASE350 deacetylation

The deacetylation assay was also performed using the smaller bench-scale ASE350 accelerated solvent extractor (Dionex, Sunnyvale, CA, USA) in the same configuration as for the dilute acid pretreatment. A 66 mL zirconium cell was used as a reaction cell. It was filled with 4.0 g of biomass and 40 mL of 0.2% (w/w) NaOH. The dry solids loading for the deacetylation pretreatment step was approximately 10% (w/w). This step consisted of a 5 min heating period followed by a 30 min static time at 80 °C. The cell temperature was then brought to 100 °C before an acid rinse step with 67 mL of 0.1% (w/w) sulfuric acid (to neutralize the solid to be able to rinse it efficiently with DI water), followed by a water rinse step with 133 mL of DI water. The liquor was collected in a 250 mL glass bottle. At the end of the assay, the liquor in the glass bottle was transferred quantitatively to a 250 mL volumetric flask that was brought to volume with DI water.

An aliquot of the liquor was transferred in separate 50 mL plastic tubes and stored in a refrigerator until further analysis. An aliquot of the liquor was hydrolyzed at 4% (w/w) sulfuric acid for 1 h at 121 °C in an autoclave and then neutralized, filtered, and analyzed for total monomeric sugars and organic acids. The washed solid was kept in the zirconium cell and stored in a refrigerator until its dilute acid pretreatment. The pretreatment was performed on the washed solid as described above, except that 1.25% sulfuric acid was used to compensate for the water contained in the washed solid (data not shown).

### ZipperClave reactor acid pretreatment

Large-scale sorghum pretreatment experiments were conducted in the ZipperClave reactor (Autoclave Engineers, Erie, PA, USA) as previously described [34]. 65.0 g dry weight of sorghum biomass was loaded into the sample container with a 1% (w/w) solution of H_2_SO_4_ to achieve a 25% solids loading (w/w). The biomass and acid solution were mixed and set for 5 min prior to pretreatment. The canister was then loaded into the ZipperClave reactor and sealed. Steam was injected using an impeller with lifting edges anchored to the bottom to ensure even heating and mixing of the contents within a removable canister during the reaction. The ZipperClave reactor was equipped with an electrical heating blanket set at the reaction temperature to lessen steam condensation due to heat losses through the reactor wall. Temperature was measured using two thermocouples located in the top and bottom of the reactor. Three reaction temperatures 140, 155, 170 °C for 10 min were used representing severities (*R*_o_) of 2.18, 2.62, and 3.06, respectively. After pretreatment, the reactor was depressurized for 20–40 s and vented steam and volatized components recovered as condensate. After the canister was removed, the agitator was rinsed to recover attached solids. The pretreated slurry, recovered from the canister, condensate, and rinsate liquors, were analyzed using standard NREL LAPs [35].

Overall mass recovery after pretreatment was 107.6 ± 2.2%, suggesting a higher bias for solids recovery. Glucose and xylose yields were normalized to 100% solids recovery to adjust for this bias in solids recovery. Total sugar yield for the control corn stover samples was 85.6 ± 3.0 g/g, demonstrating that both pretreatment and enzymatic hydrolysis processes provided robust results.

### Enzymatic hydrolysis assay

For all pretreated samples, the enzymatic hydrolysis assay was performed following the procedure of [7], which is based on the NREL LAP *enzymatic hydrolysis of lignocellulosic biomass* [36]. Briefly, the washed pretreated solid was incubated at 10% (w/w) dry solids with the enzyme (Cellic CTec2, Novozymes, Bagsvaerd, Denmark), the citrate buffer, and DI water for 5 days at 48 °C. The enzyme loading was 20 mg/g total biomass. At the end of the assay, the slurry was transferred quantitatively to a 100 mL volumetric flask and brought to volume with DI water. An aliquot of the flask was then filtered and analyzed for monomeric sugars (Additional files 2, 3, 4, 5).

### Data analysis

The raw analytical data were collected and reduced using Microsoft Excel. The open source statistical software R [37] was used to merge the Microsoft Excel files to further analyze and plot the data. All expressions of statistical significance are at the level of 5% (with *α* = 0.05) assuming independent normal distribution of errors. The statistical assessment of the total sugar yield has only been done on the WT and SM feedstocks because they are made of more replicates and are the most distinctive analyzed feedstocks (Additional files 6, 7, 8, 9).

## Additional files

**Additional file 1.** Tabular data on total sugar yields. **Table S1.** ASE350 dilute acid pretreatment yields—NU and DE calculations. **Table S2.** ASE350 PT-EH yields, with and without deacetylation, DE-NL and DE-LO calculations. **Table S3.** Zipperclave PT yields–DE calculation.

**Additional file 2.** Primary experimental data for ASE350 optimal temperature experiments.

**Additional file 3.** R script to calculate overall sugar yields from ASE350 optimal temperature data using DE method.

**Additional file 4.** R script to calculate overall sugar yields from ASE350 optimal temperature data using NU method.

**Additional file 5.** Primary experimental data for ASE350 deacetylation experiments.

**Additional file 6.** R script to calculate overall sugar yields from ASE350 deacetylation data using DE-LO method.

**Additional file 7.** R script to calculate overall sugar yields from ASE350 deacetylation data using DE-NL method.

**Additional file 8.** Primary experimental data for Zipperclave experiments.

**Additional file 9.** R script to calculate overall sugar yields from Zipperclave data.

## Abbreviations

ADF: acid detergent fiber
ADL: acid detergent lignin
*bmr*: *brown midrib*
DA: dilute acid
DE: denominator
DI: deionized
EH: enzymatic hydrolysis
eDOM: enzymatically digestible organic matter
LAP: laboratory analytical procedure
LO: loss of the released carbohydrates by deacetylation
NL: no loss of the released carbohydrates by deacetylation
NDF: neutral detergent fiber
NREL: National Renewable Energy Laboratory
NU: numerator
PT: pretreatment
SM: stacked mutant
TS: total solids
VS: organic matter (volatile solids)
WT: wild type
