# Supplementary material for: Improved sugar yields from biomass sorghum feedstocks: comparing low-lignin mutants and pretreatment chemistries
Source: Biotechnol Biofuels. 2016 Nov 21;9:251. doi: 10.1186/s13068-016-0667-y (PMC5117605; doi:10.1186/s13068-016-0667-y)
Supplement: Supplementary file 1 — Additional file 1. Tabular data on total sugar yields. Table S1. ASE350 dilute acid pretreatment yields—NU and DE calculations. Table S2. ASE350 PT-EH yields, with and without deacetylation, DE-NL and DE-LO calculations. Table S3. Zipperclave PT yields–DE calculation. [file 13068_2016_667_MOESM1_ESM.docx]

**Supplementary tables**

**Table S1. Total sugar yield (g/g) of sorghums with dilute acid (DA) pretreatment (PT) on the ASE350 reactor and enzymatic hydrolysis.**

|  | Glucose and xylose yield from structural carbohydrates (NU) | | | | Glucose and xylose yield from  structural and non-structural carbohydrates (DE) | | | |
| --- | --- | --- | --- | --- | --- | --- | --- | --- |
| Dilute acid pretreatment temperature | 150°C | 160°C | 170°C | 180°C | 150°C | 160°C | 170°C | 180°C |
| Wild type | 0.733  ±0.029 | 0.841  ±0.027 | 0.829  ±0.001 | 0.730  ±0.055 | 0.787  ±0.023 | 0.873  ±0.022 | 0.864  ±0.000 | 0.784  ±0.044 |
| Stacked mutant | 0.809  ±0.011 | 0.866  ±0.012 | 0.829  ±0.007 | 0.733  ±0.010 | 0. 847  ±0.009 | 0. .893  ±0.010 | 0.863  ±0.006 | 0.786  ±0.008 |
| *bmr6* mutant | 0.834  ±0.039 | 0.921  ±0.009 | 0.877 | 0.755 | 0.869  ±0.030 | 0.937  ±0.007 | 0.902 | 0.807 |
| *bmr12* mutant | 0.802  ±0.013 | 0.874  ±0.016 | 0.846 | 0.754 | 0.833  ±0.011 | 0.894  ±0.013 | 0.870 | 0.793 |

The uncertainty corresponds to 95% confidence interval of the mean

**Table S2. Total sugar yield (g/g) of the sorghums with or without deacetylation on the ASE350 reactor and enzymatic hydrolysis.**

|  | Glucose and xylose yield from structural and non-structural carbohydrates, and no-loss recovery of carbohydrates solubilized during deacetylation  (DE-NL) | | Glucose and xylose yield from structural and non-structural carbohydrates, and loss recovery of carbohydrates solubilized during deacetylation  (DE-LO) | |
| --- | --- | --- | --- | --- |
| Dilute acid pretreatment temperature | 150°C | 160°C | 150°C | 160°C |
| **Without deacetylation** | |  |  |  |
| Wild type | 0.787  ±0.023 | 0.873  ±0.022 | 0.787  ±0.023 | 0.873  ±0.022 |
| Stacked mutant | 0.847  ±0.009 | 0.893  ±0.010 | 0.847  ±0.009 | 0.893  ±0.010 |
| *bmr6* mutant | 0.869  ±0.031 | 0.937  ±0.007 | 0.869  ±0.031 | 0.937  ±0.007 |
| *bmr12* mutant | 0.833  ±0.011 | 0.894  ±0.013 | 0.833  ±0.011 | 0.894  ±0.013 |
| **With deacetylation** |  |  |  |  |
| Wild type | 0.858  ±0.015 | 0.889  ±0.008 | 0.680  ±0.016 | 0.711  ±0.009 |
| Stacked mutant | 0.904  ±0.013 | 0.921  ±0.008 | 0.723  ±0.012 | 0.739  ±0.007 |
| *bmr6* mutant | 0.915  ±0.015 | 0.974  ±0.068 | 0.714  ±0.017 | 0.770  ±0.063 |
| *bmr12* mutant | 0.883  ±0.005 | 0.910  ±0.001 | 0.741  ±0.002 | 0.766  ±0.005 |

The uncertainty corresponds to 95% confidence interval of the mean

**Table S3. Total sugar yield (g/g) of sorghums with dilute acid (DA) pretreatment (PT) on the ZipperClave reactor and enzymatic hydrolysis.**

|  | Glucose and xylose yield from  structural and non-structural carbohydrates (DE) | | |
| --- | --- | --- | --- |
| Dilute acid pretreatment temperature | 140°C | 155°C | 170°C |
| Wild type | 0.429  ±0.034 | 0.485  ±0.034 | 0.585  ±0.016 |
| Stacked mutant | 0.515  ±0.023 | 0.587  ±0.034 | 0.690  ±0.009 |

The uncertainty corresponds to 95% confidence interval of the mean
